# Mechanisms of Metabolic Reprogramming Regulating Immunosuppression in the Gastric Cancer Tumor Microenvironment

**DOI:** 10.3390/biom16010160

**Published:** 2026-01-16

**Authors:** Wenting Dong, Xuepeng Qian, Honglin Liu, Jinhai Huo, Weiming Wang

**Affiliations:** Institute of Chinese Materia Medica, Heilongjiang Academy of Chinese Medicine Science, Harbin 150036, China

**Keywords:** gastric cancer, immune microenvironment, metabolic reprogramming, *Helicobacter pylori*, molecular subtypes, therapeutic strategies

## Abstract

Immunotherapy, especially immune checkpoint inhibitors (ICIs), has become one of the core therapeutic approaches in cancer in recent years. It demonstrates remarkable efficacy in the treatment of melanoma and lung cancer. Conversely, its use in treating gastric cancer (GC) is not associated with considerable benefits. The high heterogeneity of GC and the tumor microenvironment (TME) may directly influence this phenomenon. This review focuses on the correlation between *Helicobacter pylori* (*H. pylori*) infection, gastric physiology, and molecular subtype-specific induction pathways, with emphasis on the unique metabolic features of GC. It explores the connection of *H. pylori* infection, gastric physiologic functions, and molecular subtype-specific induction mechanism of GC with the special metabolism of GC. It also explains the relationship between immune metabolic reprogramming and the suppressive TME in GC. Crucially, we summarize emerging therapeutic strategies targeting metabolic vulnerabilities. Furthermore, we explore the potential of subtype-guided metabolic therapies to overcome the challenges of the immunosuppressive tumor microenvironment in GC.

## 1. Introduction

Internationally, gastric cancer (GC) is one of the most frequent malignancies of the digestive system [1]. Data from the latest GLOBOCAN statistics show that about 1.089 million new cases of GC occur annually, with 769,000 deaths annually. It places GC in the fifth position for new cases and the fourth position for mortality rates globally. The 5-year survival rate of patients with metastatic GC is still below 20%, despite great accomplishments in diagnosis and therapy technology [2]. Additionally, the rates of both recurrence and metastasis lie between 60–70% [3,4]. Modern therapy approaches, such as immunotherapy, targeted therapy, or neoadjuvant chemotherapy, face obstacles related to resistance to drugs and poor immune response. One critical element is the immunosuppressive status of the tumor microenvironment (TME) [5,6]. GC cells make use of metabolic reprogramming to dynamically remodel the TME. It makes immunotherapy ineffective by impairing immune cell function directly. Besides cancer cells, the TME is composed of numerous stromal cells (such as fibroblasts or endothelial cells), infiltrating immune cells, extracellular matrix and several signaling chemicals [7]. TME eventually shifts towards an immunosuppressive phenotype when the tumor undergoes adaptive remodeling which supports malignant growth [8].

Metabolic reprogramming refers to the process by which cells adapt to specific environmental demands by altering their metabolic pathways [9]. This metabolic reprogramming can be traced back to Warburg’s observations in the 1920s [10]. He discovered that cancer cells were characterized by their robust glycolytic metabolic activities despite sufficient oxygen availability [11]. This hypothesis was initially termed as metabolic reprogramming of energy metabolism but is actually more far-reaching in that it alters the TME. Metabolic reprogramming alters the processing of nutrients in such a way that the flux of metabolites translates into tumor cell proliferation, tumor cell migration, and immune evasion [12]. Moreover, metabolic reprogramming involves the secretion of growth factors, cytokines, or metabolites by tumor-associated stromal cells, which alters the local microenvironmental and physicochemical properties. Metabolic wastes, ROS, or local hypoxia contribute to the impaired effector functions of immune effector cells while conferring a growth advantage to immunosuppressive cells [13].

Recently, immune checkpoint inhibitor-based immunotherapies have emerged as a key approach for reigniting the body’s own antitumor immune responses, with great success achieved in lung cancer, skin cancer [14,15], or other solid tumors by promoting immune cell reconstitution and harnessing the immune system to effectively combat tumors [16]. However, GC remains one of the many malignancies that have failed to achieve similar therapeutic progress with immune checkpoint inhibitors (ICIs) [17]. This difference clearly indicates that the unique biologic properties of GC hinder effective therapeutic targeting of immunotherapy interventions, as well as the complexity of the immunosuppressive tumor microenvironment present in GC [18]. Indeed, the GC tumor microenvironment is characterized by a high degree of heterogeneity driven by malignancy-specific factors, including *Helicobacter pylori* (*H. pylori*) infection, the variability of gastric pH levels, or the nutrient fluctuation, with the combined influence of such factors collectively promoting the development of a tumor microenvironment driven by metabolic properties [19,20]. These properties include substantially aberrantly activated glycolysis, upregulated glutamine metabolism, and reprogrammed lipid synthesis. These metabolic properties drive cancer cell growth and survival even in host-unfavorable conditions, further contributing to compromised effector immune cell potency or reduced immune cell accumulation by promoting the accumulation of immunosuppressive cell levels driven by factors such as nutrient scarcity, accumulation of immunosuppressive metabolites, or oxidative stress [21,22]. This clearly hinders the therapeutic potency of ICIs.

In conclusion, it is important to better appreciate the metabolic rewiring also present in GC, which is overcoming the limitations of current immunotherapeutic strategies. This review extensively explores the process of immune evasion through metabolic crosstalk between tumor cells of GC and immune cells. Special emphasis is placed on three distinctive components of GC: *H. pylori* infection, gastric acidity, and availability of nutrients. Furthermore, we analyze metabolic heterogeneity in cells of the four subtypes of GC. Crucially, we evaluate recent approaches to exploit metabolic weaknesses in GC, and discuss the potential of subtype-guided interventions to overcome the challenges of the immunosuppressive tumor microenvironment.

## 2. Metabolic Reprogramming in the Gastric Cancer Tumor Microenvironment

### 2.1. Metabolic Characteristics of Gastric Cancer Cells

**Figure 1:**
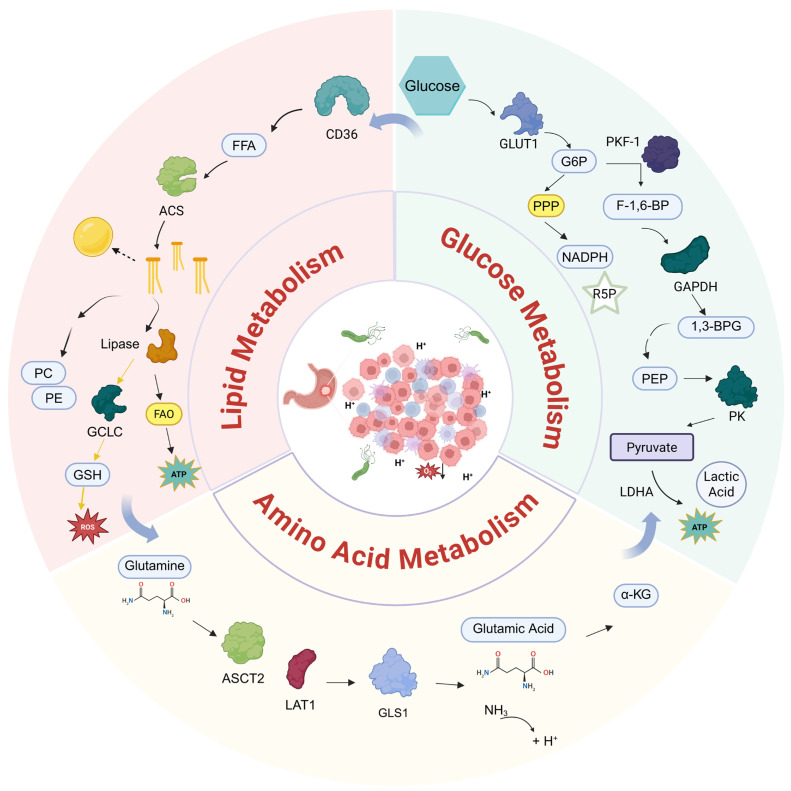
The three metabolic reprogramming pathways in gastric cancer cells. This schematic illustrates how gastric cancer cells rewire glucose, lipid, and amino acid metabolism to support malignant growth and adapt to the tumor microenvironment (TME). Glycolytic reprogramming: Driven by hypoxia and *H. pylori*, upregulation of GLUT1 and HK2 accelerates glycolysis. The Warburg effect diverts pyruvate to lactate via PKM2 and LDHA, acidifying the TME and suppressing OXPHOS, while the PPP branch supports biosynthesis and antioxidant defense. Lipid metabolic reprogramming: The *H. pylori* virulence factor CagA upregulates CD36 to enhance fatty acid uptake. These lipids fuel β-oxidation (FAO) for energy or support membrane synthesis via phospholipid production. Amino acid metabolic reprogramming: Glutamine metabolism is upregulated via GLS1 to replenish the TCA cycle (anaplerosis) and neutralize intracellular acidity through ammonia production. Crucially, glutamine metabolism is tightly coupled with CD36-mediated lipid uptake to maintain metabolic homeostasis. FFA, free fatty acid; CD36, fatty acid translocase; GLUT1, glucose transporter 1; PKF-1, phosphofructokinase-1; G6P, glucose-6-phosphate; PPP, pentose phosphate pathway; F-1,6-BP, fructose-1,6-bisphosphate; NADPH, nicotinamide adenine dinucleotide phosphate; R5P, ribose-5-phosphate; GAPDH, glyceraldehyde 3-phosphate dehydrogenase; 1,3-BPG, 1,3-bisphosphoglycerate; PEP, phosphoenolpyruvate; PK, pyruvate kinase; LDHA, lactate dehydrogenase A; ATP, adenosine triphosphate; ACS, acyl-CoA synthetase; PC, phosphatidylcholine; PE, phosphatidylethanolamine; GCLC, glutamate–cysteine ligase catalytic subunit; GSH, glutathione; FAO, fatty acid oxidation; ROS, reactive oxygen species; ASCT2, alanine–serine–cysteine transporter 2; LAT1, L-type amino acid transporter 1; GLS1, glutaminase 1; α-KG, α-ketoglutarate; NH3, ammonia.

The cancer cells in the stomach undergo robust rewiring of metabolism in order to satisfy their bioenergetic and biosynthetic requirements. This chapter highlights three primary pillars of this rewiring, which include glucose metabolism, lipid metabolism, and amino acid metabolism, and which altogether serve as promoters of tumorigenesis and immune evasion. The metabolism profile is schematically represented in Figure 1.

#### 2.1.1. Glucose Metabolic Reprogramming in Gastric Cancer Cells

Cancer cells frequently exhibit glycolytic reprogramming, a crucial component of tumor metabolic reprogramming [23]. Tumor cells alter glucose metabolism to satisfy high proliferation and energy needs in hypoxic and nutrient-deficient TME conditions, which speeds up the synthesis of ATP, albeit with efficiency significantly lower than oxidative phosphorylation (OXPHOS) [24]. Aerobic glycolysis is quite active in solid gastric malignancies, such as primary gastric lymphomas and benign gastric schwannomas [25].

By upregulating glucose transporters, GC cells improve extracellular glucose uptake by upregulating glucose transporter 1 (GLUT1), the primary transmembrane protein facilitating basal glucose transport [26]. Hexokinase 2 (HK2), the rate-limiting enzyme that catalyzes the first step of glycolysis, converts glucose to glucose-6-phosphate (G6P) [27]. Glycolysis is started by this process, which also provides metabolic intermediates for further metabolic branching. GC cells preferentially break down G6P by glycolysis, even in the presence of oxygen-sufficient TME circumstances. Pyruvate is then produced by pyruvate kinase M2 (PKM2), a critical enzyme governing the final step of glycolysis to generate ATP [28]. In contrast to normal cells, this pyruvate rarely enters mitochondria for OXPHOS. Recent evidence highlights that this restriction is mechanistically dictated by defects in the mitochondrial pyruvate carrier complex, specifically the downregulation of MPC1 and MPC2 subunits, which serve as the major gatekeepers for mitochondrial pyruvate entry [29,30]. Furthermore, loss of ALDH4A1, an important modifier that maintains the integrity of the MPC complex, has been documented in several malignancies to destabilize the complex and further promote this glycolytic phenotype [31]. As a result of this functional loss of the ALDH4A1-MPC axis, pyruvate flux is shunted toward Lactate dehydrogenase A (LDHA), leading to significant lactate accumulation and TME acidification [32]. This process not only rapidly produces ATP to meet immediate energy demands but also maintains NAD+ regeneration required for glycolysis by consuming NADH, ensuring its sustained operation. Moreover, G6P has been shown to enter the pentose phosphate pathway (PPP), thereby generating ribose-5-phosphate (R5P) and NADPH, which are indispensable for the synthesis of nucleic acids and lipids [33]. By controlling pyruvate production rates and balancing energy and anabolic synthesis to sustain proliferation, PKM2 indirectly affects the diversion of G6P to the PPP [34]. In order to speed up glucose metabolism, tumor growth causes local hypoxia, which further upregulates important glycolytic enzymes such as GLUT1 and HK2 [34,35]. At the same time, metabolic enzymes have the ability to directly affect mitochondria, increasing their reliance on glycolysis [36]. By binding to voltage-dependent anion channel (VDAC), a key gatekeeper controlling the passage of metabolites across the outer mitochondrial membrane, HK2 suppresses signals of mitochondrial apoptosis and directs pyruvate toward glycolysis. OXPHOS efficiency is decreased by PKM2’s direct inhibition of the mitochondrial electron transport chain [37,38,39].

#### 2.1.2. Lipid Metabolic Reprogramming in Gastric Cancer Cells

De novo lipid synthesis (DNL), exogenous absorption, and fatty acid oxidation (FAO) are coordinated by lipid metabolic reprogramming to satisfy cellular needs for signaling, energy supply, and biomembrane formation [40]. One essential lipid metabolic process that sets GC cells apart from other cancer types is CD36-mediated exogenous lipid uptake.

GC is highly dependent on the chronic inflammatory microenvironment induced by *H. pylori* infection [41]. *H. pylori* increases the expression of the fatty acid transporter CD36 (a fatty acid translocase that facilitates the uptake of exogenous lipids), which improves the efficiency of GC cells’ uptake of lipoproteins and free fatty acids (FFAs) [42]. In particular, the phosphorylation of NF-κB in gastric mucosal cells is triggered by the *H. pylori* virulence factor CagA, a major oncoprotein injected into host cells [43]. NF-κB moves into the nucleus, attaches itself to the CD36 promoter, and immediately increases the expression of CD36 [44]. Ingested FFAs are activated by acyl-CoA synthase (ACS) and converted into triglycerides (TG). These are stored in lipid droplets as an energy reserve during hypoxia [45]. When hypoxia in the tumor center reduces glycolytic efficiency and glucose becomes scarce, TG is broken down by lipase into FFAs. These FFAs then generate substantial ATP through β-oxidation (FAO). GC cells that divide quickly also require substantial phospholipid synthesis to make new cell membranes [46]. FFA absorption mediated by CD36 supplies essential building blocks for the production of phospholipids [47]. Key constituents of novel membrane architectures are phosphatidylcholine (PC) and phosphatidylethanolamine (PE), which are produced by phosphatidylcholine synthase and phosphatidylethanolamine synthase, respectively [48].

#### 2.1.3. Amino Acid Metabolic Reprogramming in Gastric Cancer Cells

Tumor cells alter nutrition use patterns by amino acid metabolic reprogramming to boost proliferation, counter oxidative damage, and improve malignant characteristics [49,50]. The distinct glutamine metabolic reprogramming that GC cells display in response to the combined impacts of *H. pylori* infection, mucosal hyperacidity, and dietary variations works in concert with exogenous lipid uptake.

Through virulence factors, *H. pylori* infection directly controls important glutamine metabolism molecules to improve the efficacy of extracellular glutamine uptake [51]. It works in concert with glutaminase 1 (GLS1), the mitochondrial enzyme responsible for converting glutamine into glutamate, to boost the generation efficiency of α-ketoglutarate (α-KG) [52]. The adaptive functional adaptation of glutamine metabolism in GC cells is driven concurrently by the physiological milieu of fasting glucose deprivation and the high acidity of the gastric mucosa [53,54]. GLS1 expression is upregulated in GC cells when intracellular lactate builds up in an acidic environment, which catalyzes glutamine breakdown [55]. In order to compensate for the energy deficit induced by decreased glycolytic efficiency in acidic conditions, the produced α-KG enters the tricarboxylic acid (TCA) cycle and OXPHOS pathways to produce ATP [55,56,57]. The NH_3_ produced by glutamate deamination can neutralize intracellular H^+^, maintaining pH homeostasis. This prevents acidity-induced apoptosis [58].

GC cells tightly couple glutamine metabolism with CD36-mediated exogenous lipid uptake. Glutamine-derived α-KG is converted into citrate via the TCA cycle. Upon cytoplasmic transport, ATP citrate lyase (ACLY) catalyzes its conversion to acetyl-CoA, providing key precursors for the synthesis of PC, PE and TG [32,59,60,61]. Conversely, Glutamate-cysteine ligase catalytic subunit (GCLC) activity is increased by exogenous fatty acids that CD36 uptakes, which encourages the synthesis of glutathione (GSH). In order to prevent ROS-mediated GLS1 inactivation and preserve glutamine metabolic equilibrium, GSH scavenges ROS produced during glutamine breakdown [62,63].

### 2.2. Metabolic Reprogramming of Immune Cells

In the GC TME, metabolic stress is caused by the competing nutrition consumption of cancer cells as well as adaptive changes in invading immune cells [64]. Even though these modifications help immune cells survive, they frequently result in diminished anticancer activity, which eventually creates an immunosuppressive milieu (Table 1).

#### 2.2.1. CD8^+^ T Cells

Ferroptosis and tryptophan deficiency are two important processes that inhibit CD8^+^ T cell activity [65,66,67]. The overexpression of indoleamine 2,3-dioxygenase 1 (IDO1), a rate-limiting enzyme that degrades the essential amino acid tryptophan, by tumor cells in the TME accelerates the breakdown of tryptophan, resulting in local depletion [68]. A lack of tryptophan activates general control nonderepressible 2 (GCN2) kinase, a cellular sensor of amino acid starvation, which inhibits mTOR translational activity, lowers IL-2 and granzyme B (GZMB) secretion, halts CD8^+^ T cell proliferation and differentiation, and reduces their cytotoxic potential [69,70]. Moreover, GC’s low-glucose, high-lactate TME suppresses mTOR signaling, which compels CD8^+^ T cells to upregulate CD36 and carnitine palmitoyltransferase 1A (CPT1A), the rate-limiting enzyme for fatty acid oxidation (FAO), in order to transition to FAO as an energy source [71,72]. This adaptive response results in iron-dependent lipid peroxidation-mediated cell death when the iron levels in the GC TME are elevated.

After being absorbed through CD36, polyunsaturated fatty acids (PUFAs) are esterified by acyl-CoA synthetase long-chain family member 4 (ACSL4), an enzyme essential for lipid metabolism and sensitivity to ferroptosis, and integrated into the cell membrane. Through the Fenton reaction, these esters stimulate the generation of excessive ROS under Fe^2+^ enrichment, leading to severe membrane phospholipid peroxidation and the production of cytotoxic 4-hydroxy-2-nonenal (4-HNE). Low expression of the lipid repair enzyme glutathione peroxidase 4 (GPX4) in dysfunctional CD8^+^ T cells prevents them from repairing lipid peroxidation damage, which eventually causes cell membrane rupture and death [73,74,75].

#### 2.2.2. Tumor-Associated Macrophages

The main metabolic mediators driving tumor-associated macrophages (TAMs) towards an immunosuppressive M2 phenotypic transition are lactic acid and tryptophan depletion. Lactic acid is taken up by target cells by monocarboxylate transporters (MCTs). It decreases prolyl hydroxylase (PHD), thereby stabilizing hypoxia-inducible factor-1α (HIF-1α), a master transcriptional regulator of cellular adaptation to hypoxic stress. This factor triggers the expression of immunosuppressive genes, including vascular endothelial growth factor (VEGF), and arginase-1 (Arg1) [76,77,78]. This factor also binds to the G-protein-coupled receptor GPR81, thereby increasing levels of intracellular cAMP. This factor triggers M2 gene expression, including Arg1, by protein kinase A (PKA). These pathways induce an M2 phenotypic transition in TAMs. These pathways result in angiogenesis in the tumor and an inhibition of T cell activation [79,80].

The level of IDO1 expression is constantly enhanced in both TAMs and GC cells [81]. IDO1 has the ability to metabolize tryptophan, an activator of T cells. Its metabolite, Kynurenine (Kyn), also serves as an endogenous ligand of the Aryl Hydrocarbon Receptor (AhR), a ligand-activated transcription factor involved in immune modulation, hence activating it [82]. The activated AhR enhances the immunosuppressive properties of TAMs. When it translocates into the nucleus, it binds to Aryl hydrocarbon receptor nuclear translocator (ARNT), forming dimers. Then, it binds to the promoter regions of target genes, hence triggering the transcription of immunosuppressive genes [83].

#### 2.2.3. Regulatory T Cells

Regulatory T cells (Tregs) depend on OXPHOS to preserve functional homeostasis. This contrasts with effector T cells, which prefer glycolysis [84]. Treg cells actively uptake lactate through high expression of monocarboxylate transporter 1 (MCT1). Lactate is converted into pyruvate, which then enters the TCA cycle for efficient energy production [85].

Lactate also suppresses PHD activity, which stops HIF-1α from being ubiquitinated and degraded [86]. As a result, it accumulates in the nucleus, increasing forkhead box P3 (FOXP3), the master transcription factor determining Treg development and function, to maintain the suppressive effect of Tregs [87,88]. Furthermore, Kyn buildup in the TME triggers AhR activation, which causes CD4^+^ T cells to differentiate into Tregs and futher increases FOXP3 expression [89].

Additionally, *H. pylori* infection promotes Tregs’ glutamine metabolism. Tregs absorb more extracellular glutamine and convert it to α-KG, which replenishes the TCA cycle to support ATP generation via OXPHOS [90,91]. This constantly activates glutamine metabolism while maintaining Tregs’ survival and immunosuppressive cytokine release in the low-glucose microenvironment [90,92]. Concurrently, Jumonji domain-containing protein 3 (JMJD3) activity is maintained by α-KG, via histone demethylation at the FOXP3 promoter. This procedure stabilizes the expression of FOXP3 by releasing transcriptional repression on it [93]. As a result, Tregs facilitate tumor immune evasion by continuously inhibiting CD8^+^ T cell activation in the inflammatory milieu of GC [94,95].

#### 2.2.4. Natural Killer Cells

Toll-like receptor 4 and 9 (TLR4/9) on the surface of natural killer (NK) cells recognize *H. pylori* virulence factors and activate the NF-κB pathway [96]. In NK cells, NF-κB inhibits important OXPHOS enzymes and increases pyruvate dehydrogenase kinase 1 (PDK1), which lowers glycolytic rates and OXPHOS efficiency. This considerably impairs their capacity to identify and eliminate GC cells [97,98].

Moreover, lactate can hinder NK cell activity in a number of ways, and NK cells are susceptible to extremely acidic extracellular conditions [99]. It hinders efficient tumor cell identification by downregulating the expression of NKG2D and DNAM-1 on NK cell surfaces [100]. It inhibits intracellular signaling by lowering intracellular pH, which decreases the synthesis of the important effector cytokine interferon-γ (IFN-γ) [101,102]. Lactate abrogates NK cell cytotoxicity by suppressing the mTOR pathway. This suppression reduces the production of perforin and granzymes and finally hinders the polarization of cytotoxic granules and the formation of synapses [101,103].

## 3. Metabolic Crosstalk in the TME

**Figure 2:**
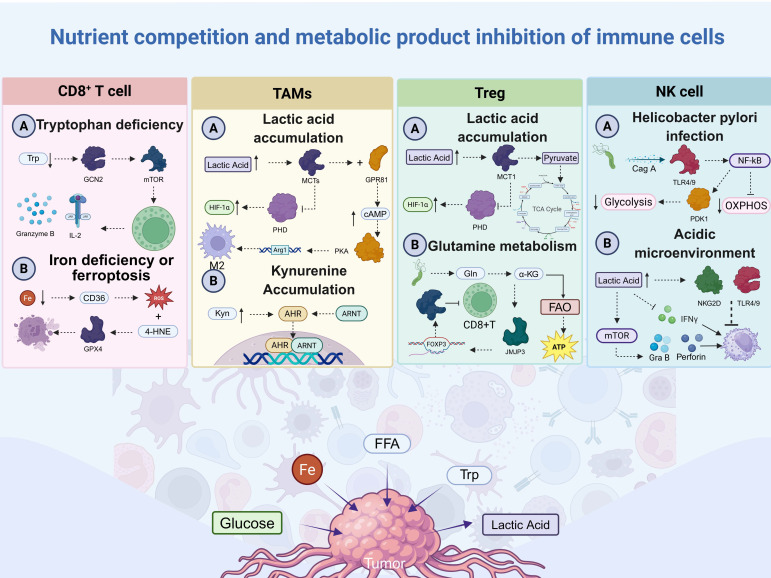
Nutrient competition and metabolic interactions between gastric cancer cells and immune populations. The nutrient-deficient (glucose, tryptophan, iron, and FFAs) and metabolite-rich (lactate and kynurenine) context favors the expansion of gastric cancer cells. CD8^+^ T cells: An impaired function phenotype results from the depletion of tryptophan through IDO1, glucose deprivation, and ferroptosis mediated by lipid peroxidation. TAMs: The accumulation of lactate and kynurenine stimulates the macrophage transition to an immunosuppressive M2 phenotype through the HIF-1α and AhR pathways. Tregs: The uptake of lactate and kynurenine stabilizes the FOXP3 transcription factor and supports the differentiation of suppressive Tregs. NK cells: The impaired cytotoxic functions of NK cells are mediated by. FFA, free fatty acid; Trp, tryptophan; GCN2, general control nonderepressible 2; mTOR, mammalian target of rapamycin; IL-2, interleukin-2; Fe, iron; CD36, fatty acid translocase; ROS, reactive oxygen species; 4-HNE, 4-hydroxy-2-nonenal; GPX4, glutathione peroxidase 4; TAMs, tumor-associated macrophages; MCTs, monocarboxylate transporters; HIF-1α, hypoxia-inducible factor-1α; PHD, prolyl hydroxylase; GPR81, G-protein-coupled receptor 81; cAMP, cyclic adenosine monophosphate; Arg1, arginase-1; PKA, protein kinase A; Kyn, kynurenine; AhR, aryl hydrocarbon receptor; ARNT, aryl hydrocarbon receptor nuclear translocator; MCT1, monocarboxylate transporter 1; TCA, tricarboxylic acid; Gln, glutamine; α-KG, α-ketoglutarate; FAO, fatty acid oxidation; ATP, adenosine triphosphate; FOXP3, forkhead box P3; JMJD3, Jumonji domain-containing protein 3; CagA, cytotoxin-associated gene A; NF-κB, nuclear factor-kappa B; TLR4/9, Toll-like receptor 4/9; OXPHOS, oxidative phosphorylation; PDK1, pyruvate dehydrogenase kinase 1; NKG2D, natural killer group 2 member D; IFNγ, interferon-gamma; Gra B, granzyme B.

The metabolic conflict between tumor cells and immune cells is the cause of the immunosuppressive characteristics of the GC TME [104]. Important substrates including glucose, glutamine, and iron are competitively used by tumor cells, depriving immune cells of nutrients [105]. They simultaneously release lipids, lactic acid, and metabolites produced from amino acids, all of which directly impair the activity of effector immune cells [106]. Ultimately, immune cells create a metabolic milieu that promotes tumor growth by adapting to metabolic stress at the price of anticancer function [92] (Figure 2).

### 3.1. Glucose

GLUT1 is extensively expressed by GC cells, which have a much greater capacity to glucose uptake than immune cells. It is important to clarify that this increased uptake targets glucose from the systemic circulation and interstitial fluid within the TME, rather than direct absorption of dietary glucose from the gastric lumen. As a result, the TME’s glucose concentrations are significantly lower [107]. Concomitantly, they use glycolysis to generate excessive lactate, which is then exported into the TME through monocarboxylate transporter 4 (MCT4) [108]. Lactate further inhibits CD8^+^ T cell glycolysis in the acidic environment, causing them to convert to ineffective fatty acid oxidation (FAO) [108]. However, Tregs restrict glycolysis through PDK1 to sustain metabolic demands via OXPHOS, giving them a survival advantage in low-glucose environments [109].

### 3.2. Glutamine

Glutamine, a critical source of nitrogen and carbon for protein synthesis and TCA cycle anaplerosis, is a core nutrient contested by tumors and immune cells [110]. Glutamate transporters such as alanine–serine–cysteine transporter 2 (ASCT2) and L-type amino acid transporter 1 (LAT1) are upregulated in GC cells, giving them a complete uptake advantage [111]. T cell function is significantly suppressed by glutamine deficiency. It concurrently forces T cells to transition to OXPHOS metabolism and initiates T cell death caused by endoplasmic reticulum stress. The ability of IL-2 and GZMB is diminished [90,112].

### 3.3. Iron Competition

By upregulating iron absorption proteins such as transferrin receptor (TfR1), GC cells actively collect iron resources from the TME and store them as ferritin to support their growth [113]. At the same time, they release ferritin to inhibit the expression of the macrophage iron export protein ferroportin (FPN), which results in the retention of iron within macrophages and increases the expression of heme oxygenase-1 (HO-1) [114,115]. This dual action causes M2 polarization and decreases phagocytic capability.

Iron competition is exacerbated by *H. pylori* infection. In the stomach mucosa and the TME, *H. pylori* directly competes with immune cells for iron by expressing iron acquisition proteins such as *H. pylori* DppA (HpDppA) and ferric citrate transporter A (FecA) [116]. Its virulence factor CagA also triggers the stomach mucosa’s NF-κB–HIF-1α pathway, which causes GC cells to overexpress TfR1 and prevents immune cells from releasing iron, resulting in a serious iron distribution imbalance [117,118].

### 3.4. Lipid Competition

By suppressing adipocyte lipolysis, GC cells release angiopoietin-like 4 (ANGPTL4), which drastically lowers the amount of FFAs available in the TME [119]. Concurrently, *H. pylori* infection increases the efficiency of tumor cell FFAs absorption by activating CD36 signaling [120]. In contrast, low or suppressed CD36 expression makes it difficult for T cells and macrophages to efficiently acquire lipids [44,121]. Tregs can survive on a small amount of lipids, but CD8^+^ T cells are more vulnerable to ferroptosis because they do not produce enough GPX4, which makes them unable to withstand the damage caused by 4-HNE-mediated lipid peroxidation [122,123].

## 4. Unique Metabolic Characteristics of Gastric Cancer

Tumor cells and immune cells engage in metabolic competition, which gives the GC TME its immunosuppressive characteristics. Important substrates including glucose, glutamine, and iron are competitively used by tumor cells, depriving immune cells of nutrients. They simultaneously produce metabolites generated from amino acids, lipids, and lactic acid, all of which directly impair the activity of effector immune cells [124,125,126]. Immune cells adapt to metabolic stress at the expense of their antitumor function. This ultimately creates a metabolic environment conducive to tumor progression.

### 4.1. Helicobacter Pylori Infection

Roughly 80% of GC is associated with chronic infection by *H. pylori* [127]. It is a distinguishing pathogenic factor for GC. It is involved in metabolic reprogramming through its virulence factors. The metabolic interaction of *H. pylori* with host cells is a distinguishing characteristic of GC over other cancers [128].

*H. pylori* changes the metabolism of cancer cells through specific virulence factors. It activates the NF-κB pathway, increasing the expression of crucial glycolytic genes and glycolytic activity. An active *H. pylori* infection also demonstrates regulatory effects on the tricarboxylic acid cycle dynamics. It starts by inhibiting the activity of the tricarboxylic acid cycle to induce glycolysis [33,129]. As the infection progresses, the TCA cycle is repurposed to supply biosynthetic precursors that the tumor needs to keep growing [130,131]. In lipid metabolism, *H. pylori* upregulates CD36 expression, promoting FFA uptake. In contrast, vacuolating cytotoxin A (VacA) has been shown to augment lipid influx through the activation of membrane channels. Consequently, these processes result in the accumulation of intracellular lipids and the activation of oncogenic pathways [132]. In relation to amino acid metabolism, *H. pylori* upregulates ASCT2 and GLS1, thereby enhancing glutamine catabolism and supplying tumor cells with energy and biosynthetic precursors. At the same time, it increases IDO1 activity to change the metabolism of tryptophan, which in turn triggers AhR signaling to cause Tregs differentiation [89].

*H. pylori* affects both innate and adaptive immunity through particular metabolic manipulations at the immunoregulatory level [133]. The *H. pylori* virulence factor lipopolysaccharide (LPS) triggers innate immunity by upregulating PDK1 to prevent glycolysis, activating NK cell NF-κB signaling through TLR4, and inducing macrophage Arg1 expression to encourage M2 polarization [79,97,98]. By stabilizing Tregs’ immunosuppressive functions through the Trp–Kyn–AhR axis and depleting iron resources in the TME through iron transporters such as FecA, *H. pylori* contributes to adaptive immunity by creating an immunosuppressive TME [113].

### 4.2. Anatomy and Physiological Microenvironment of Stomach

The stomach’s physiological environment and anatomical structure produce a unique microenvironment that is favorable to GC. Strong acidity and sporadic changes in nutrient availability are characteristics of the GC tumor microenvironment [134]. By altering the physicochemical characteristics and substrate availability in the microenvironment, the characteristics listed have a substantial impact on cellular metabolic states and act as basic background conditions for the development of GC.

The highly acidic gastric lumen exerts specific regulatory effects on metabolism [135]. By increasing glutamine metabolism and upregulating pH-regulating proteins such as carbonic anhydrase IX (CAIX), GC cells preserve intracellular homeostasis [136]. They provide growth advantages in acidic environments by using GLS to catalyze the generation of ammonia for intracellular H^+^ neutralization and α-KG to replenish the TCA cycle for energy supply [137]. Conversely, by suppressing phosphofructokinase-1 (PFK1) activity to prevent effector T cell glycolysis and lowering NKG2D receptor expression on NK cells, the acidic environment progressively reduces antitumor immunity [108,138].

Nutrient concentrations in the TME fluctuate significantly due to periodic gastric emptying [139]. High metabolic flexibility has evolved in GC cells, allowing them to change their main metabolic pathways in response to different dietary circumstances [140]. They enhance glycolysis by upregulating GLUT1 and HK2 during postprandial hyperglycemia [141,142]. CPT1-regulated β-oxidation and CD36-mediated FFAs uptake sustain the energy supply during fasting [143]. By changing the local metabolite composition, this metabolic flexibility affects other cell survival in addition to maintaining tumor cell growth in the face of nutritional variations. For example, glutamine depletion in the TME is caused by fasting tumor cells upregulating ASCT2. T cells undergo apoptosis as a result of an endoplasmic reticulum unfolded protein response (UPR) [18,89].

### 4.3. Metabolic Heterogeneity in Gastric Cancer Molecular Subtypes

Depending on the molecular basis of the tumor, these key drivers have variable effects on different types of GC. Highly diverse molecular subtypes have evolved as a result of this targeted selection by etiology and microenvironment. These subtypes each employ distinct metabolic or phenotypic reprogramming strategies to respond to environmental stress. Gastric tumors were divided into four molecular subgroups by The Cancer Genome Atlas (TCGA) in 2014: chromosomal instability (CIN), microsatellite instability (MSI), genomic stability (GS), and Epstein–Barr virus (EBV)-positive [144]. These four molecular subtypes show very different metabolic and phenotypic reprogramming patterns in addition to having different genetic bases and signaling pathways.

## 5. Metabolic and Phenotypic Reprogramming in Gastric Cancer Subtypes

**Figure 3:**
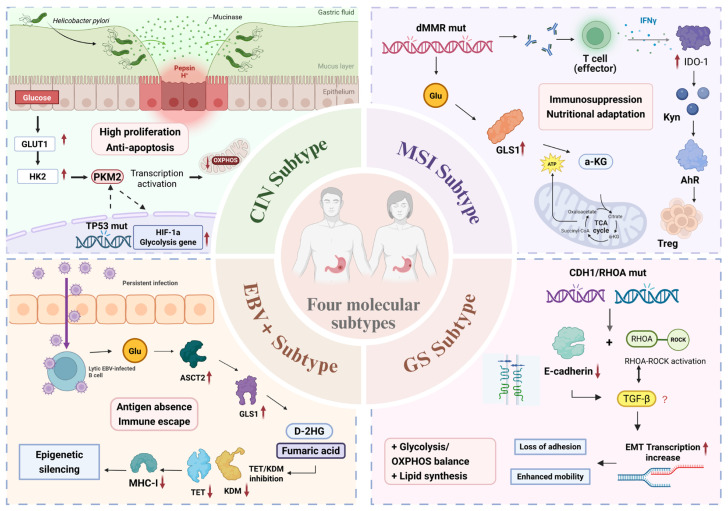
Subtype-specific mechanisms of metabolic reprogramming and immune evasion in gastric cancer. This schematic illustrates the distinct drivers, core pathways, and functional outcomes of the four primary molecular subtypes in gastric cancer. CIN Subtype: *H. pylori* infection and TP53 mutation are the primary drivers. They activate the PI3K–AKT–mTOR axis and PKM2, enhancing aerobic glycolysis. This metabolic shift promotes rapid tumor proliferation and resistance to apoptosis; MSI Subtype: Mismatch repair deficiency causes a high mutational burden. This triggers an IFN-γ-dependent immune response that upregulates IDO1. IDO1 activation depletes tryptophan and produces kynurenine, suppressing effector T cells and promoting Treg activity. This subtype also adapts to nutrient fluctuations via flexible glutamine metabolism; EBV-positive subtype: Epstein–Barr virus latent infection hijacks glutamine metabolism. This leads to the accumulation of oncometabolites (D-2HG, fumarate). These metabolites inhibit epigenetic enzymes, causing widespread gene silencing. A key silenced target is the MHC class I antigen presentation machinery, enabling immune evasion; GS subtype: This subtype is defined by morphological reprogramming, not canonical metabolic changes. Mutations in CDH1 and RHOA disrupt cell adhesion and activate the RHOA–ROCK axis. This drives an epithelial–mesenchymal transition (EMT), resulting in diffuse infiltration, early peritoneal metastasis, and poor prognosis. CIN, chromosomal instability; *H. pylori*, *Helicobacter pylori*; *TP53*, tumor protein p53; GLUT1, glucose transporter 1; HK2, hexokinase 2; PKM2, pyruvate kinase M2; OXPHOS, oxidative phosphorylation; HIF-1α, hypoxia-inducible factor-1α; MSI, microsatellite instability; dMMR, deficient mismatch repair; Glu, glutamine; GLS1, glutaminase 1; ATP, adenosine triphosphate; α-KG, α-ketoglutarate; TCA, tricarboxylic acid; IFN-γ, interferon-gamma; IDO1, indoleamine 2,3-dioxygenase 1; Kyn, kynurenine; AhR, aryl hydrocarbon receptor; Treg, regulatory T cell; EBV, Epstein–Barr virus; ASCT2, alanine–serine–cysteine transporter 2; D-2HG, D-2-hydroxyglutarate; TET, ten–eleven translocation; KDM, lysine demethylase; MHC-I, major histocompatibility complex class I; GS, genomically stable; *CDH1*, cadherin 1; RHOA, ras homolog family member A; ROCK, Rho-associated protein kinase; TGF-β, transforming growth factor-beta; EMT, epithelial–mesenchymal transition.

GC has been shown to display considerable heterogeneity, and this is particularly observed when compared with the main molecular subtypes. The main molecular subtypes are generated based on completely different biological backgrounds, including specific pathogenic factors and genetic mechanisms (Table 2). GC cells with various characteristics display completely different trends in interaction with the tumor microenvironment, thus demonstrating specific regulation mechanisms (Figure 3).

### 5.1. CIN Subtype

The chromosomal instability (CIN) subtype is the most common form of GC, accounting for approximately 50% of all cases. Tumors of this subtype tend to arise at the gastroesophageal junction or in the cardia [144]. The rate of *H. pylori* infection is often above 85%. The patients usually present with chronic inflammation of the gastric mucosa and abnormal acid secretion [145]. Metabolic reprogramming in the CIN subtype is characterized by highly active glycolysis. Its development is closely associated with *TP53* mutations and is further influenced by the background of *H. pylori* infection.

In the CIN subtype, the *TP53* mutation rate exceeds 70%. It increased glycolysis dependence in concert with *H. pylori*. CagA activates NF-κB signaling [146]. Mutated p53 loses transcriptional repression of GLUT1 and HK2. This results in significantly higher expression of glucose transporters and hexokinase compared to other subtypes. This facilitates highly efficient glucose uptake by tumor cells within the gastric tissue [147,148]. Concurrently, mutant p53 cannot properly activate peroxisome proliferator-activated receptor gamma coactivator 1-alpha (PGC1α) [149]. OXPHOS and mitochondrial biogenesis are disrupted. The inhibition of the mitochondrial electron transport chain by *H. pylori* CagA blocks the oxidative energy supply, forcing cells to switch to glycolytic dependence [150]. In addition, enhanced glycolysis interacts with the mitochondrial ROS leakage induced by the acidic gastric environment. Mutated p53 forms a complex with nuclear factor erythroid 2-related factor 2 (Nrf2), upregulating antioxidant genes (such as GCLC and superoxide dismutase 2 [SOD2]) to counteract ROS accumulation [151]. This creates a vicious cycle that not only keeps metabolic homeostasis in the acidic microenvironment, but also supports cell proliferation.

Beyond *TP53* mutations, EGFR/MET amplification rates are 40–50%, further enhancing the positive feedback of glycolysis through the PI3K–AKT–mTORC1 axis [152]. AKT phosphorylation inhibits prolyl hydroxylase domain protein 2 (PHD2), stabilizes HIF-1α, and activates glycolytic genes like *GLUT1/3*, *HK2*, and *LDHA* [153]. Simultaneously, AKT phosphorylates HK2’s Ser473, improving its ability to bind to VDAC. By preventing cytochrome c release and increasing glycolytic start efficiency, this prevents acidity-induced mitochondrial apoptosis and achieves metabolic reprogramming in addition to anti-apoptosis. Additionally, ribosomal protein S6 kinase beta-1 (S6K1) is activated by mTORC1 to support nuclear localization and c-Myc translation [154,155]. By binding to the E-box element on the LDHA promoter, c-Myc increases the efficiency of lactate synthesis and strengthens the “Warburg effect” [156,157].

Building on these regulatory mechanisms, chromosomal instability causes CIN subtypes to proliferate, which further solidifies the glycolytic phenotype through epigenetic control and PKM2-mediated metabolic diversion [158]. *H. pylori* CagA can activate Src kinase and promote PKM2 serine phosphorylation because of its continuously high PKM2 expression [159]. Glycolytic intermediates build up and the pentose phosphate pathway is diverted by low PKM2 activity, which produces NADPH to scavenge ROS and R5P for nucleic acid synthesis [160]. More importantly, phosphorylated PKM2 forms a PKM2–HIF-1α–JMJD3 complex by entering the nucleus through importin α5 [161]. This complex lifts the transcriptional inhibition of glycolytic genes by mediating H3K27me3 demethylation through JMJD3. This mechanism identifies PKM2 as a crucial node that connects epigenetic control with metabolic reprogramming [162,163].

### 5.2. MSI Subtype

The MSI-type results from changes in DNA mismatch repair (MMR) genes, accounting for 22% of all GC cases [164]. It mostly happens in the distal stomach and has a lower *H. pylori* infection rate (40–50%) [165]. Its core regulation involves immune suppression mediated by tryptophan metabolism and nutritional adaptation driven by glutamine metabolism.

The activation mechanism of the MSI subtype’s tryptophan metabolism differs from that of the CIN subtype. Given its lower *H. pylori* infection rate, the high expression of IDO1 is not directly induced by *H. pylori* [166,167]. Instead, it is associated with an immune-active tumor microenvironment shaped by a high mutation burden. Specifically, DNA mismatch repair defects enhance neoantigen presentation, thereby activating adaptive immune responses and elevating IFN-γ levels [168]. Through the JAK–STAT1 pathway, IFN-γ induces STAT1 nuclear translocation and binding to the IDO1 promoter, sustaining continuous activation of the Trp–Kyn axis [169]. Consequently, local tryptophan supply becomes progressively limited due to sustained IDO1 activation, impairing effector T cell function [133]. Simultaneously, accumulated metabolites promote Tregs activation and TAMs polarization toward the M2 phenotype, collectively reinforcing immune evasion [170].

Moreover, in this subtype, there is flexible glutamine metabolism to compensate for nutritional variations in the gastric body [171]. Owing to the lack of *H. pylori*-CagA-mediated ASCT2/LAT1 expression, its efficiency in transporting exogenous glutamine is decreased, thus improving endogenous glutamine synthesis [172]. When fasting, under nutrient-deprived conditions, AMPK/STAT3 signaling pathways increase glutamine synthetase gene expression. GC cells preferentially metabolize glutamates derived from TCA cycle activity to provide endogenous glutamine synthesis to satisfy cellular demands [173,174]. This endogenously generated glutamine serves to maintain TCA cycle activity and regulation of pH balance [175]. ASCT2 levels increase postprandially; hence, its utilization efficiency for exogenous glutamine increases [176]. At the same time, glucose-derived NADPH influences glutamine catabolism to provide carbon source components for biosynthesis [177,178].

### 5.3. EBV-Positive Subtype

The EBV-positive subtype accounts for approximately 9% of all GC cases, which mostly affect younger men and develop in the stomach’s fundus or body [179]. EBV-positive infection raises the risk of GC by almost ten times, according to studies [180]. This subtype’s main characteristic is that the virus actively manipulates the metabolism of the host cell, employing certain metabolites to promote widespread epigenetic silencing and eventually creating an immune-evading milieu [181]. The virus’s predominant role in carcinogenesis is shown by the fact that this mechanism is distinct from the tryptophan metabolism-mediated immunosuppression of the MSI subtype and the glycolysis-dependent immunosuppression of the CIN subtype.

In metabolic reprogramming, viral components generated during latent EBV-positive infection serve as key upstream factors [182]. EBV-encoded small RNAs (EBERs) upregulate ASCT2 expression to improve glutamine absorption by activating the NF-κB and PI3K–AKT signaling pathways [183]. By triggering downstream kinase signaling, the latent membrane protein 2A (LMP2A) increases GLS1 activity, but it also encourages glutamine breakdown [184]. By greatly increasing cellular glutamine metabolism, this synergistic improvement creates a large supply of substrates for the subsequent aberrant buildup of metabolites such as fumarate and D-2-hydroxyglutarate (D-2HG) [185]. Particularly in EBV-positive GC cells, fumarate and D-2HG build up under long-term viral control [186]. These metabolites function as competitive inhibitors of α-KG, suppressing the activity of DNA demethylase ten–eleven translocation (TET) and histone demethylase lysine demethylase (KDM). This results in genome-wide epigenetic reprogramming: Inhibitory histone marks accumulate extensively, causing chromatin compaction [144,187]. A distinctive CpG island methylator phenotype (CIMP) is formed when CpG islands in gene promoter regions experience hypermethylation at the same time. When combined, these mechanisms inhibit the expression of many genes that are involved in function [188].

Antigen presentation is one of the main targets of this epigenetic silencing. The genes encoding MHC class I molecules exhibit severe expression loss due to promoter hypermethylation. This renders them incapable of effectively presenting viral antigens to CD8^+^ T cells [189,190,191]. As a result, immunological escape occurs because the immune system is unable to identify GC cells. This represents a fundamental difference from the pathways that suppress T cell function in the MSI subtype.

### 5.4. GS Subtype

The genomic-stable (GS) subtype accounts for approximately 20% of all GC cases [192]. It is characterized by mutations in genes like *CDH1*, *ARID1A*, and *RhoA.* Histologically, it primarily presents as diffuse-type Lauren GC, with around 25% of cases developing at the cardia or gastroesophageal junction [193,194]. This subtype’s primary characteristic is morphogenetic reprogramming, which is triggered by alterations in cell adhesion and signaling pathways rather than usual metabolic reprogramming. Its ability to disrupt cell connections and modify the cytoskeleton allows cancer cells to diffusely infiltrate, which sets it apart from other subtypes.

The GS subtype’s widespread phenotype is mostly caused by synergistic mutations in the *RHOA* and *CDH1* genes [195]. About 40–60% of patients have inactivating *CDH1* mutations, which cause E-cadherin function to be lost, intercellular junctions to be disrupted, and nuclear translocation of β-catenin to activate genes related to the epithelial-mesenchymal transition (EMT) [196,197]. At the same time, *RHOA* hotspot mutations are present in 20% to 30% of cases. Cell motility is improved by the prolonged activation of *RHOA*, which causes actin cytoskeletal contraction and reconfiguration by downstream Rho-associated protein kinase (ROCK) [192,198]. These mutations work together to greatly reduce cell adhesion and encourage migration. At the regulatory level, the RHOA–ROCK axis and TGF-β signaling pathways work in concert to promote morphologic remodeling in GS GC. The RHOA–ROCK axis directly drives motility by targeting the phospho-myosin light chain, which improves cell contraction and stress fiber production [199,200]. A mesenchymal undifferentiated state that resembles embryonic migratory characteristics is maintained by abnormal TGF-β signaling, which also upregulates EMT)transcription factors and creates a positive feedback loop with the RHOA–ROCK axis [201]. There is no distinct metabolic reprogramming in GS GC. Its metabolic changes mainly include moderately increased lipid synthesis and balanced use of oxidative phosphorylation and glycolysis [127,144]. As an adaptive reaction to high invasiveness, these alterations mainly supply energy and raw materials for membrane biogenesis and cytoskeletal remodeling.

Its distinct biological behavior and therapeutic obstacles are highlighted by the process centered on morphologic remodeling, which causes clinical hurdles in GS GC, such as ambiguous borders, early peritoneal metastasis, and poor prognosis [202,203].

## 6. Therapeutic Strategies Targeting Metabolic Reprogramming

Considering the key role of metabolic reprogramming in the shaping of the immunosuppressive TME and driving drug resistance, there is a change in the therapeutic approach from broad-spectrum cytotoxicity to precise metabolic intervention. These metabolic vulnerabilities are targeted to starve tumor cells of their much-needed nutrients, which also acts to restore a favorable metabolic landscape for antitumor immunity.

### 6.1. Targeting Glycolytic and Mitochondrial Interaction

The “Warburg effect” driven by HK2, PKM2, and LDHA acts as the primary energetic engine for gastric cancer progression. Since *H. pylori* infection initiates aerobic glycolysis via CagA and Lonp1, eradication of *H. pylori* remains the fundamental metabolic intervention to halt early-stage metabolic rewiring [204,205]. Among the enzymatic targets, HK2 serves as a critical gatekeeper. Small molecule inhibitors such as 3-Bromopyruvate (3-BrPA) and 2-Deoxy-D-glucose (2-DG) have shown efficacy in preclinical models by competitively blocking glucose flux or dissociating HK2 from mitochondrial VDACs, thereby triggering mitochondria-associated apoptosis [206,207]. In the context of drug resistance, Metformin has garnered attention for its ability to promote the degradation of the circadian regulator PER1. Disruption of this PER1-HK2 axis suppresses HK2-mediated glycolysis and has been shown to restore sensitivity to Trastuzumab in HER2-positive GC [208]. Additionally, the PPARα agonist Fenofibrate offers another strategy by activating the PPARα pathway to promote fatty acid oxidation, effectively shifting the metabolic preference away from glycolysis [209].

Downstream of HK2, PKM2 regulates the final rate-limiting step of glycolysis. Beyond its metabolic function, PKM2 translocates into the nucleus to promote HIF-1α transcription. The small molecule activator TEPP-46 can tetramerize PKM2, thus blocking its nuclear translocation and forcing a metabolic shift back to oxidative phosphorylation, thereby dampening HIF-1α-driven immunosuppression [210]. Furthermore, the proton pump inhibitor Pantoprazole (PPZ) has been repurposed as a PKM2 inhibitor; it reverses multidrug resistance by inhibiting the Akt/GSK-3β/β-catenin signaling pathway [211]. Finally, targeting LDHA is essential for neutralizing the acidic TME. Inhibitors such as Oxamate can suppress mTOR activity and induce protective autophagy [212]. Most importantly, lactate accumulation promotes the dysfunction of tumor-infiltrating lymphocytes. Tregs in the high-glycolytic TME actively take up lactate through MCT1, which induces the nuclear translocation of NFAT1 and thereby directly upregulates PD-1 expression. This high PD-1 expression leads to enhanced suppressive activity of Tregs and resistance against PD-1 blockade [85]. Targeting the LDHA-lactate axis, therefore, stands as a strategic avenue to avoid Treg hyper-activation and sensitize tumors against immune checkpoint inhibitors.

### 6.2. Lipid Metabolism and Fatty Acid Uptake as Targets

Aberrant lipid metabolism, characterized by enhanced uptake, de novo synthesis, and oxidation, supports GC metastasis and fosters an immunosuppressive TME. The fatty acid translocase CD36 is identified as a critical dependency for metastasis-initiating GC cells, wherein it activates the AKT/GSK-3β/β-catenin pathway to drive epithelial–mesenchymal transition [213]. Crucially, CD36 is also a metabolic checkpoint for MDSCs. Tumor-infiltrating MDSCs actively absorb fatty acids through CD36, resulting in lipid accumulation and enhanced capacity for immunosuppression against CD8^+^ T cells. Anti-CD36 mAb may thus have a two-way impact on tumors and MDSC: blocking tumor metastasis while preventing MDSC dysfunction and thereby maintaining antitumor immunity [214,215].

Aside from uptake inhibition, the interference with lipid biosynthesis and modifications offers other therapeutic targets. Avasimibe, an inhibitory agent of sterol O-acyltransferase 1 (SOAT1), inhibits cholesterol esterification and thus interferes with lymphangiogenesis and the growth of tumor cells due to VEGF-C downregulation [216]. Of greater importance is Avasimibe’s role in promoting cytotoxicity of CD8^+^ T cells through an enhancement of plasma membrane cholesterol concentrations. This increases TCR signaling and the formation of the immune synapse. The combination of Avasimibe and anti-PD-1 therapy was shown to be synergistically effective in models of human cancers [217]. Moreover, inhibition of the HDAC class can lead to the recalcitrant issue of lipid metabolism through the enhancement of ACSL4 and the activation of lipid peroxidation-induced ferroptosis of GC cells due to VEGF-C downregulation and *VHL* gene silencing [218]. To overcome the adaptive resistance based on the ability of cells to resort to FAO when under starvation conditions, the CPT1A inhibitor Etomoxir has been proven to inhibit the immunosuppressive function of T-MDSCs and Tregs, which depend largely on FAO for their viability [219,220].

### 6.3. Targeting Amino Acid Metabolism and Immune Tolerance

Amino acid deprivation is also an effective immune evasion strategy for GC, and modulation of these metabolic checkpoints is an approach to reverse tolerance to the immune system. IDO1 catalyzes the rate-limiting step of tryptophan conversion to KYN; this process is often upregulated due to infection with *H. pylori* via the cGAS-IRF3 pathway [221]. IDO1 has an intimate link with the immunologic checkpoints; for example, CTLA-4-positive Tregs can upregulate IDO1 proteins on dendritic cells, making IDO1 an immunosuppressive feedback loop [222,223]. The selective IDO1 inhibitor Epacadostat inhibits this process by reducing the production of KYN. Clinical trials demonstrated that administration of Epacadostat with Pembrolizumab can lead to long-lasting responses, especially when there are high tumor infiltrates [223]. Again, IDO1 inhibition also led to an improvement when targeting Claudin18.2 CAR-T as an approach to restore T cell metabolic competency within an immunosuppressive host [224].

Parallel approaches are also focused on glutamine and arginine metabolism. For glutamine, the glutaminase inhibitors, CB-839, also known as Telaglenastat, inhibit glutaminolysis, thereby triggering metabolic crisis in GCs, but overcome competition for nutrients to enable effector T cells to reestablish their use of oxidative metabolism [225]. For arginine, the new arginase dual inhibitor, OATD-02, reestablishes the concentration of L-arginine in the tumor microenvironment by simultaneously inhibiting both ARG1 and ARG2 enzymes, resulting in a reduction in polyamine production, ultimately potentiating antitumor immune responses by potently increasing T-cell infiltration in the core areas of the tumors [226]. The different strategies collectively target amino acids with the purpose of utilizing the desert of the tumor microenvironment to support an immune reaction against the cancer.

## 7. Conclusions

GC has persisted as a lethal malignancy with high diversity and a strongly immunosuppressive TME. Although significant success has been seen with the development of immunotherapy, its clinical benefits in GC are often compromised by limited T-cell infiltration and acquired resistance. As discussed in this review, the association between metabolism and the development of an immunosuppressive TME has shown that increased nutrient uptake by tumor cells leads to a nutrient-deprived microenvironment. The nutrient-deprived microenvironment fails to support effector T cells but instead favors the development of suppressive T cells.

One of the major differences between GC metabolism and other solid cancers is that GC metabolism is highly affected by the *H. pylori* infectious gastric physiological microenvironment. *H. pylori* acts as one of the major modulators of metabolism, triggering glycolysis transitions and tryptophan depletion prior to maximal carcinogenesis. This suggests that targeted therapy for GC metabolism should incorporate *H. pylori* status, where treatment to eradicate *H. pylori* serves as one of the primary metabolism-regulating interventions. In addition, the variation in nutrient levels based on gastric physiology creates a constantly changing selective pressure that mandates strong metabolic plasticity in GC cells, which can change from glycolytic to FAO, among others. This is why single-target metabolic inhibitors tend to develop adaptive resistance.

Despite the limited success of unstratified metabolic therapy approaches in trials, one main contributing factor to this would be the metabolic variability that exists between GC molecular subtypes. As discussed, the CIN subtype is addicted to glycolysis and lipogenesis, whereas the GS subtype relies heavily on stromal-driven FAO. This dichotomy indicates that a metabolic inhibitor effective in one subtype, such as FASN inhibitors for CIN, might be futile in another, such as GS. Therefore, the subtype-guided therapeutic framework proposed in this review offers a rational approach to precision medicine. Future trials should hence target specific subtypes based on metabolic patterns, such as using IDO1 inhibitors in MSI-H.

Despite such advancements, major gaps persist between harnessing concepts of metabolism to enhance survival rates. One major limitation is that current understanding relies heavily on static models, which fail to capture the dynamic metabolic alterations accompanying *H. pylori* colonization, carcinogenesis, and metastasis. Furthermore, although mechanisms within CIN/MSI subtypes have been carefully established, the metabolic interactions within GS or EBV-positive subtypes, especially those involving interactions between metabolites in regulating antigen presentation, remain relatively uninvestigated. Beyond mechanistic gaps, the systemic toxicity of metabolic inhibitors constitutes a significant hurdle, as targeting tumor-specific metabolic vulnerabilities without impairing normal tissue homeostasis requires more precise delivery systems.

To bridge these translational gaps, the future research direction should be the application of patient-derived organoids and multi-omics analyses, such as single-cell metabolomics and spatial transcriptomics, for the dynamic profiling of the TME metabolic phenotypes. Additionally, comprehensive clinical trials are crucial for the verification of distinct metabolic biomarkers in the context of the currently used subtypes. Notably, the interaction between neo-adjuvant therapy and host metabolism warrants deeper investigation; therapy-induced systemic metabolic impairments, such as glucose/lipid disorders or amino acid imbalances, may fundamentally alter nutrient availability and tumor metabolic plasticity, thereby influencing the efficacy of subsequent metabolic interventions. Ultimately, these insights should drive the design of rational combination therapies, rather than empiricism. For example, the use of lipid metabolic modifiers and anti-PD-1 therapy for the treatment of lipid-rich tumors for the purpose of reversing immune resistance.

In conclusion, the immune microenvironment in GC is influenced by metabolic reprogramming, distinct from the infectious and physiological contexts. By leveraging the power of the non-Warburg metabolic perspective and its applicability to the specific type of GC, we unlock novel therapeutic targets. Pursuing the metabolic weakness provides an actionable solution to the bottleneck issue of immune infiltration in non-immunogenic tumors and possibly the immunotherapy in patients with GC.

## Figures and Tables

**Table 1:** Summary of metabolic reprogramming characteristics and functional consequences in gastric cancer immune cells.

Cell Type	Primary Metabolic Dependency	Key Metabolic Alterations	Key Molecular Regulators	Functional Consequence
Effector T Cells (CD8^+^)	Glycolysis (Compromised) → FAO (Forced)	1. Glucose uptake ↓ (due to competition) 2. Tryptophan availability ↓ 3. Lipid peroxidation ↑	IDO1, CPT1A, CD36, ACSL4, GPX4 ↓	Impaired cytotoxicity; Ferroptosis induction; Decreased IFN-γ/GZMB production.
Regulatory T Cells (Tregs)	OXPHOS	1. Lactate uptake ↑(fuels OXPHOS) 2. Glutamine catabolism ↑	MCT1, FoxP3, AhR	Enhanced suppressive function; Stabilization of FoxP3; Increased survival in acidic/hypoxic TME.
TAMs (M2-like)	OXPHOS	1. Lactate uptake ↑ 2. Kynurenine accumulation ↑	HIF-1α, Arg1, AhR	Polarization toward immunosuppressive M2 phenotype; Promotion of angiogenesis.
NK Cells	Glycolysis/OXPHOS (Both inhibited)	1. Glycolysis ↓ 2. Mitochondrial function ↓	mTOR ↓, NF-κB (activated by *H. pylori*)	Downregulation of NKG2D; Impaired granule secretion and tumor killing.

Note: Arrows (↑/↓) indicate upregulation or downregulation of metabolic pathways/molecules in the gastric cancer TME compared to normal physiological conditions or effector phenotypes.

**Table 2:** Biological characteristics, metabolic features, and immune regulatory mechanisms of the four primary molecular subtypes of gastric cancer.

Subtype	Proportion	Primary Location	Prognosis	Key Drivers	Metabolic Features	Immune & Microenvironment Characteristics
CIN	~50%	Gastroesophageal junction/cardia	Medium	*H. pylori* (>85%), *TP53* mut, EGFR/MET amplification	Hyperactive Glycolysis (GLUT1/HK2 ↑); Lipid Synthesis ↑	Lactate accumulation; T cell exhaustion; Hypoxic TME
MSI	~22%	Distal stomach	Better	dMMR, High mutational burden	Amino Acid Dependency (Trp depletion via IDO1; Gln addiction)	High T cell infiltration (Hot tumor); IDO1-mediated immune tolerance; IFN-*γ* signaling
EBV	~9%	Fundus/Body	Medium	Latent EBV infection	Epigenetic Rewiring (D-2HG/Fumarate accumulation); Glutaminolysis ↑	Impaired antigen presentation (MHC-I ↓); “Immune-hot” but sup-pressed phenotype; PD-L1 ↑
GS	~20%	Diffuse type	Poor	*CDH1/RHOA* mut, CLDN18-ARHGAP fusion	Fatty Acid Oxidation (FAO); Balanced bioenergetics	Fibrotic/Stromal-rich TME (CAF interaction); EMT-driven invasiveness; “Immune-cold”

Note: Arrows (↑/↓) indicate the upregulation or downregulation of metabolic pathways, gene expression, or protein levels characteristic of each subtype. Abbreviations: CIN, chromosomal instability; MSI, microsatellite instability; EBV, Epstein-Barr virus; GS, genomic stability; TME, tumor microenvironment; dMMR, deficient mismatch repair; FAO, fatty acid oxidation.

## Data Availability

No new data were created or analyzed in this study. Data sharing is not applicable to this article.

## Abbreviations

The following abbreviations are used in this manuscript:

ICIs	Immune checkpoint inhibitors
GC	Gastric cancer
TME	Tumor microenvironment
*H. pylori*	*Helicobacter pylori*
OXPHOS	Oxidative phosphorylation
GLUT1	Glucose transporter 1
HK2	Hexokinase 2
G6P	Glucose-6-phosphate
PKM2	Pyruvate kinase M2
LDHA	Lactate dehydrogenase A
PPP	Pentose phosphate pathway
R5P	Ribose-5-phosphate
VDAC	Voltage-dependent anion channel
DNL	De novo lipid synthesis
FAO	Fatty acid oxidation
FFAs	Free fatty acids
ACS	Acyl-CoA synthase
TG	Triglycerides
PC	Phosphatidylcholine
PE	Phosphatidylethanolamine
α-KG	α-Ketoglutarate
GLS1	Glutaminase 1
TCA	Tricarboxylic acid
ACLY	ATP-citrate lyase
P-Cho	Phosphocholine
GCLC	Glutamate-cysteine ligase catalytic subunit
GSH	Glutathione
IDO1	Indoleamine 2,3-dioxygenase 1
GCN2	General control nonderepressible 2
GZMB	Granzyme B
CPT1A	Carnitine palmitoyltransferase 1A
PUFAs	Polyunsaturated fatty acids
ACSL4	Acyl-CoA synthetase long-chain family member 4
4-HNE	4-Hydroxy-2-nonenal
GPX4	Glutathione peroxidase 4
TAMs	Tumor-associated macrophages
MCTs	Monocarboxylate transporters
PHD	Prolyl hydroxylase
HIF-1α	Hypoxia-inducible factor-1α
VEGF	Vascular endothelial growth factor
Arg1	Arginase-1
PKA	Protein kinase A
Kyn	Kynurenine
AhR	Aryl hydrocarbon receptor
ARNT	Aryl hydrocarbon receptor nuclear translocator
Tregs	Regulatory T cells
MCT1	Monocarboxylate transporter 1
FOXP3	Forkhead box P3
JMJD3	Jumonji domain-containing protein 3
TLR4/9	Toll-like receptor 4 and 9
NK	Natural killer
PDK1	Pyruvate dehydrogenase kinase 1
IFN-γ	Interferon-γ
MCT4	Monocarboxylate transporter 4
ASCT2	Alanine–serine–cysteine transporter 2
LAT1	L-type amino acid transporter 1
TfR1	Transferrin receptor
FPN	Ferroportin
HO-1	Heme oxygenase-1
HpDppA	*H. pylori* DppA
FecA	Ferric citrate transporter A
ANGPTL4	Angiopoietin-like 4
VacA	Vacuolating cytotoxin A
LPS	Lipopolysaccharide
CAIX	Carbonic anhydrase IX
PFK1	Phosphofructokinase-1
UPR	Unfolded protein response
TCGA	The Cancer Genome Atlas
CIN	Chromosomal instability
MSI	Microsatellite instability
GS	Genomic stability
EBV	Epstein–Barr virus
Nrf2	Nuclear factor erythroid 2-related factor 2
SOD2	Superoxide dismutase 2
PHD2	Prolyl hydroxylase domain protein 2
S6K1	S6 kinase beta-1
MMR	Mismatch repair
EBERs	EBV-encoded small RNAs
LMP2A	Latent membrane protein 2A
D-2HG	D-2-hydroxyglutarate
TET	Ten–eleven translocation
CIMP	CpG island methylator phenotype
EMT	Epithelial–mesenchymal transition
